# Prevalence and determinants of gestational diabetes mellitus among pregnant women in India: an analysis of National Family Health Survey Data

**DOI:** 10.1186/s12905-024-02936-0

**Published:** 2024-02-29

**Authors:** Aditi Chakraborty, Suryakant Yadav

**Affiliations:** Department of Bio-Statistics and Epidemiology, International Institute for Population Sciences (IIPS), Mumbai, Maharashtra India

**Keywords:** Gestational diabetes, Maternal health, Public health and health disparities

## Abstract

**Background:**

Gestational diabetes mellitus (GDM) is a type of diabetes with its first recognition during pregnancy. GDM is a high-risk maternal and neonatal condition which increases the risk of Type 2 diabetes in mothers and their infants. It is essential to detect and treat GDM since its inception when mothers suffer from Type 1 diabetes while carrying the foetus during the gestational period.

**Methods:**

The study analysed individual data from the National Family Health Survey (NFHS) surveyed in 2015–2016 (4th round) and 2019–2021 (5th round) covering a total of approximately 6 lakhs and 7 lakhs women, respectively. Among them, 32,072 women in 2015–2016 and 28,187 in 2019–2021 were pregnant, of whom 180 women in 2014–2015 and 247 women in 2019–2021 had diabetes during their gestational periods, allowing the percentage prevalence calculation of GDM. The analysis of Poisson regression estimates examined the socioeconomic and demographic risk factors for GDM among pregnant women.

**Results:**

The overall prevalence of GDM in women showed an increase from 0.53% in 2015–16 to 0.80% in 2019–20 at the national level, and a similar increase in many states of India was witnessed, with a few exceptions. The GDM prevalence has shown a gradient over age, with a low prevalence in 15–19- and 25–29-year-olds and the highest prevalence in 40–44-year-olds. Concerning the rural and urban divide, its prevalence in both urban and rural areas has increased from 0.61 to 0.85% and 0.51 to 0.78% between 2015 and 16 and 2019–21. The results of the Poisson regression analysis reveal that older adults with high Body Mass Index (BMI), thyroid disorder, and heart disease have a greater risk of GDM among pregnant women in India. The states of Kerala, Meghalaya, and Goa show a high prevalence of GDM.

**Conclusion:**

The low prevalence of GDM may not be clinically significant but has negative repercussions on the mother and her child cannot be overlooked. Thus, it is essential to curb GDM since its inception and save a generation ahead from the risk of diabetes and other diseases.

**Supplementary Information:**

The online version contains supplementary material available at 10.1186/s12905-024-02936-0.

## Background

Pregnancy, a life altering event, entails physiological changes with inherent health risks. GDM, a prevalent disorder [1], manifests during pregnancy [2]. Unlike Type 1 diabetes, where the pancreas provides minimal or no insulin, type 2 diabetes involves insulin resistance. This resistance, linked with hormonal imbalances and varying insulin secretion, amplifies during pregnancy, affecting women’s hormones, metabolism, and increasing the risk of developing GDM.

Women who develop GDM may also have preexisting beta cell dysfunction in the pancreas as a result of insulin resistance. This beta cell dysfunction limits the sensitivity of the pancreas for insulin secretion. It impairs glucose intolerance which may lead to enduring GDM. Although GDM is associated with maternal and foetal complications for index pregnancy, women with GDM after pregnancy and delivery face a ten-fold higher risk of developing type 2 diabetes [3]. Essential Preventive measures, follow-up and intense screening for women with GDM before and after delivery are crucial for impending Type 2 diabetes risk in the Indian population [4].

### Pathophysiology associated with GDM

GDM heightens the risk of obstetrical complications and adverse foetal outcomes, including preeclampsia, caesarean delivery, stillbirth, macrosomia and hypoglycaemia [5–8]. It is a prominent risk factor for type 2 diabetes mellitus, impacting both mothers and newborns. Additionally, its recurrence correlates with later life hypertension and cardiovascular diseases [9].

For more than a century, it has been acknowledged that pregnancy with diabetes can have adverse effects on mothers and their foetuses [10]. Numerous studies emphasise elevated neonatal mortality and foetal deaths in women with high GDM prevalence. Historically, GDM, linked to type 2 diabetes possess life threatening complications during pregnancy, underscoring its role as a risk factor for successful pregnancy [11].

By the 1950s, the term “gestational diabetes” was thought to be a transient condition adversely affecting foetal conditions [12]. The term ‘gestational diabetes mellitus’ (GDM) was introduced by O’Sullivan in 1961 [13]. Also, Researchers reveal a link between the extent of glucose intolerance during pregnancy and the post pregnancy diabetes risk. They introduced statistical criteria for interpreting Oral glucose tests (OGTTs), establishing cut-off values based on two standard deviations. In the 1980’s, these values were adjusted to modern glucose measurement methods, shaping the contemporary definition of gestational diabetes [14].

**Fig. 1:**
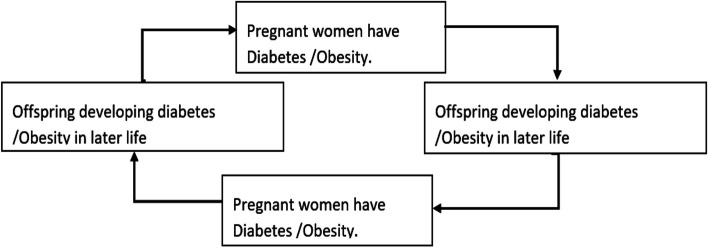
Vicious cycle showing intergenerational transmission of GDM

Research in humans and animals has demonstrated that in utero, the environmental disruption can deform organ structure and metabolism, causing subtle physiological changes. This early metabolic resistance contributes to the premature onset of diabetes, leading to related issues such as cardiovascular diseases and obesity [15]. The hypothesis of the “foetal origin of adult disease” proposes that gestational programming is influenced by adult health and disease [16], Transmits metabolic imprinting from obese and diabetic intrauterine environments to subsequent generations [17]. Offspring exposed to gestation glucose intolerance face an elevated lifelong risk [18]. The pieces of literature show a vicious cycle of the hazards of glucose intolerance, explaining an increase in obesity, GDM, and Type 2 diabetes in the past several decades [19, 20]. The presence of GDM spurs this vicious cycle where affected mothers have offspring prone to future metabolic diseases, perpetuating GDM in subsequent generations, as shown in Fig. 1. To break this vicious cycle, preventive measures against Type 1 and Type 2 diabetes should start during the intrauterine period.

### Indian scenario

The global rise in diabetes prevalence is more pronounced in developing countries, surpassing developed nations***.*** Literatures on GDM in India are less studied, potentially due to the Government prioritising NCDs particularly cardiovascular diseases. In Bangalore, a study conducted in the public health facilities, an IT Hub urban city in southern India, reported GDM prevalence below 1% [21]. A Rohtak district study on 607 females revealed 7.1% GDM prevalence [22]. In Kashmir, the extreme northern part of India, the GDM prevalence was 3.8% [23]. Tamil Nadu, an economically advanced southern state, showed varying urban (17.8%), semi-urban (13.8%) and rural (9.9%) GDM rates [20]. These findings underscore GDM’s significant prevalence alongside its wide regional variation in the country.

In India, the pioneering study by Seshiah and others (2006) led to the adoption of the ‘Diabetes in Pregnancy Study Group in India (DIPSI)’ program as the diagnostic standard for GDM [24]. GDM is a growing public health concern globally and in India, where diabetic women are projected to reach 313.3 million by 2040 [25]. Over five million Indian pregnant women are estimated to be affected by GDM, constituting 16.2% of total live births (20.9 million) in 2015 had some form of hyperglycaemia. Among these 16.2% live births, with 85.1% attributed to GDM, with around four million women affected, India is now considered as the diabetes capital of the world [26]. The risk factors include high BMI, nutritional intake, long-term contraceptive use, physical inactivity, multimorbidity, high glycaemic index (GI) food and hyperthyroidism [27–29]. The high prevalence of GDM associated with many risk factors points to unwarranted consequences on women’s health and their newborns.

Acknowledging the repercussions of GDM among mothers and their children, this study aims to examine the socioeconomic and demographic determinants of GDM in India. Specifically, the objectives of the study are (1) to examine the change in the percentage prevalence of GDM in women during 2015–2016 and 2019–2021, (2) to examine socioeconomic and demographic risk factors for GDM, and (3) to examine the regional variation in the prevalence of GDM in India.

## Data and methods

### Data source

This study analysed the 4th round (2015–2016) and 5th round (2019–2021) of the NFHS. It is a large-scale, multi-round cross-sectional survey conducted and coordinated by the International Institute of Population Sciences (IIPS), Mumbai, under the aegis of the Ministry of Health and Family Welfare (MOHFW), Government of India (GOI). Technical assistance in both of these surveys was provided mainly by ICF, USA and some other organisations on specific issues. The funding for the different rounds was provided by United States Agency for International Development (USAID), Department for International Development (DFID), the Bill and Melinda Gates Foundation, United Nation Children’s Fund (UNICEF), United Nations Population Fund (UNFPA), and MOHFW, GOI.

### Data description

The NFHS adopted a multistage stratified systematic sampling design and provides detailed information on a representative sample of 699,686 and 724,115 women aged 15–49 years in 2015–16 and 2019–21, respectively, at the national level. In this sample, 30,832 and 26,350 women were pregnant in 2015–16 and 2019–21, respectively. Pregnant women in both surveys were asked for their diabetic status. Of these women, 162 in 2015–16 and 208 in 2019–21 reported ‘Yes’ for GDM disease. The remaining women reported ‘No’ for the same. We excluded 348 and 219 pregnant women who reported their diabetic status as ‘not knowing’ in 2015–16 and 2019–21, respectively. The study posits its findings based on a sample of pregnant women who were aware of their diabetic status. Effectively, the sample of pregnant women we considered for analytical purposes in the study was 30,484 and 26,131 in 2015–16 and 2019–21, respectively. Further, we considered 28 states of India for the analysis, and Union territories were excluded because of the low sample size.

### Description of variables

The variable of interest in the study is the prevalence of GDM in pregnant women. The number of pregnant women who reported gestational diabetes, i.e., numerator, was divided by the total number of pregnant women, i.e., denominator, to calculate the prevalence of GDM. To examine the risk factors for GDM, the dependent variable considered was the prevalence of GDM in pregnant women who were aware of their diabetic status. The study considered predictor variables that can be broadly categorised into three categories, i.e., demographic, socioeconomic, and individual characteristics. The demographic variables are age and place of residence; the socioeconomic variables are religion groups, caste groups, level of education, and wealth index; and the individual variables are BMI categories, alcohol consumption, tobacco consumption, thyroid disorder, hypertension, heart disease, and children ever born.

### Statistical analysis

Descriptive statistics of the unadjusted prevalence of GDM in 2015–16 and 2019–21 by socioeconomic and demographic variables at the national level were calculated and presented with sample size. Unadjusted prevalence for the states for the year 2015-16 and 2019-21 was calculated. The null hypothesis of the same or specified distribution between GDM and age, religion, caste, and place of residence was tested against an alternate hypothesis of a different distribution between categories of GDM (Yes and No) using the chi-square test. A Poisson regression model was performed to examine the risk factors for GDM and to estimate the adjusted prevalence of GDM among pregnant women in India after controlling for a set of variables. Both Deviance goodness of fit and Pearson goodness of fit was performed to check the goodness of fit for the regression model.

## Result

Table 1 shows the descriptive statistics of the prevalence of GDM in 2015–16 and 2019–21. A total of 162 women (0.53%) out of 30,484 pregnant women and 208 (0.80%) out of 26,131 pregnant women in 2015–16 and 2019–21, respectively, were diagnosed with GDM. The mean age of pregnant women was 24.61 years with a standard deviation of 4.85 years in 2015–16 and 24.85 years with a standard deviation of 4.82 years in 2019–21.

**Table 1:** Descriptive statistics of the prevalence of Gestational Diabetes Mellitus (GDM)

	Variables	Sample	Mean	Std. Dev	Min.	Max.
NFHS-4 (2015–16)	GDM	162				
	Age	30,484	24.60	4.84	15	49
	BMI	30,314	21.81	4.40	.26	455.07
	Children ever born	30,484	1.05	1.05	0	3
NFHS-5 (2019–21)	GDM	208				
	Age	26,131	24.85	4.82	15	49
	BMI	26,123	22.22	9.18	.31	493.59
	Children ever born	26,131	.97	1.00	0	3

Source: Own calculations using NFHS (2015–16 and-2019–21) data

The average number of children ever born to these pregnant women was 1.05 children with a standard deviation of 1.06 in 2015–16 and 0.97 children with a standard deviation of one in 2019–21. The average BMI of pregnant women was 21.81 kg/m^2^ with a standard deviation of 4.40 in 2015–16 and 22.22 kg/m^2^ with a standard deviation of 9.18 in 2019–21 (Table 1).

Table 2 shows the prevalence of GDM by socioeconomic and demographic characteristics, such as age groups, religions, castes, and types of places of residence, in the Indian population in 2019–21. The prevalence of GDM increased from the lowest value of 0.48% in the age group of 15–19 years to the highest value of 3.91% in the age group of 40–44 years. The age group 45–49 years showed a prevalence of GDM of 2.44%, showing a plunge in gradient compared to that in younger age groups. The prevalence of GDM among different religious groups reveals that Christian women show the highest prevalence of GDM at 1.24%, followed by Muslim women (1.09%) and Hindu women (0.69%). The prevalence of GDM was higher among women in the upper caste than among women in the lower caste, i.e., Scheduled Castes (SCs) and Scheduled Tribes (STs). The prevalence of GDM was higher in women residing in urban areas than in those residing in rural areas; however, the association of GDM with caste (*P* value = 0.644) and type of residence (P value = 0.641) based on the chi-square test was not statistically significant during 2019–21 (Table 2).

**Table 2:** The prevalence of GDM across age groups, religious groups, caste groups and type of residence during 2015-16 and 2019-21

**Selected background characteristics/Prevalence over time**	**2015-16**	**2019-21**	**Between 2015-16 and 2019-21**	
	**Prevalence of GDM (%)**	**Prevalence of GDM (%)**	**Absolute change in prevalence of GDM**	**Relative change in prevalence of GDM**
	**(1)**	**(2)**	**(3)**	**(4)**
**Age groups**	***	***		
**15-19**	0.34	0.48	0.14	41.18
**20-24**	0.44	0.52	0.08	18.18
**25-29**	0.55	0.87	0.32	58.18
**30-34**	0.84	1.21	0.37	44.05
**35-39**	0.72	2.10	1.38	191.67
**40-44**	2.17	3.91	1.74	80.18
**45-49**	1.59	2.44	0.85	53.46
**Religion categories**	**	*		
**Hindus**	0.51	0.69	0.18	35.29
**Muslims**	0.76	1.09	0.33	43.42
**Christians**	0.34	1.24	0.90	264.71
**others**	0.46	0.69	0.23	50.00
**Caste groups**				
**SCs**	0.54	0.84	0.30	55.56
**STs**	0.43	0.82	0.39	90.70
**Others**	0.90	0.72	-0.18	-20.00
**Place of residence**	*			
**Urban**	0.61	0.85	0.24	39.34
**Rural**	0.51	0.78	0.27	52.94

Source: Own calculations using NFHS (2015-16 and 2019-21) data

Note: Prevalence is per hundred women; ***, **, * represents level of significance at 1%, 5% and 10%, respectively. N=30484 during 2015-16 and 26131 during 2019-21

Column (3) is supported by supplementary figures: FigA1, FigA2, FigA3, FigA4

Table 2 also illustrates the dynamic changes in GDM prevalence in the Indian women between 2015 and 16 and 2019–21, across demographic categories. The prevalence increased notably across age-groups, for instance, from 0.34 to 0.48% in the 15–19 age-group and from 0.72 to 2.1% in the 35–39 age-group. Similarly, across religious groups, the highest relative change was observed in Christian women (264.71%). While GDM prevalence increased for SCs and STs women, there was a decline in the upper caste women (− 20.00%). Urban areas experienced a surge from 0.61 to 0.85%, surpassing rural areas’ increase from 0.51 to 0.78%. The Chi square test indicates that the caste category does not exhibit statistically significant associations (*P* values of 0.272 during 2015–16 and 0.644 during 2019–21) during both the years. Whereas, place of residence does not show a statistically significant association during 2019–21(*P*-value-0.641).

Table 3 shows the prevalence of GDM in the states of India in 2015–16 and 2019–21. The prevalence of GDM in pregnant women in India was estimated to be 0.53% in 2015–16 and increased to 0.80% in 2019–21. Most of the states of India show an increase in the prevalence of GDM, with an exception in Arunachal Pradesh showing a decline in the prevalence from 1.61% in 2015–16 to 0.87% in 2019–21. Similarly, a decline in GDM was noticeable in other states of India, such as Bihar (0.92% in 2015–16 to 0.60% in 2019–21), Gujarat (0.46% in 2015–16 to 0.35% in 2019–21), Kerala (3.41% in 2015–16 to 3.06% in 2019–21), Manipur (0.15% in 2015–16 to 0.00% in 2019–21), Tamil Nadu (1.2% in 2015–16 to 0.96% in 2019–21), and Telangana (1.57% in 2015–16 to 0.69% in 2019–21). A striking increase in the prevalence of GDM was noticeable in Meghalaya, where the prevalence increased from 0.15% in 2015–16 to 2.33% in 2019–21. A similar increase in the prevalence of GDM was found in Tripura (0.00% in 2015–16 to 1.64% in 2019–21), Nagaland (0.25% in 2015–16 to 1.14% in 2019–21), Karnataka (0.42% in 2015–16 to 1.81% in 2019–21), Goa (2.86% in 2015–16 to 4.88% in 2019–21), and Andhra Pradesh (0.00% in 2015–16 to 0.63% in 2019–21) (Table 3).

**Table 3:** Prevalence of GDM across the states of India

States of India	Unadjusted Prevalence of GDM (2015-2016)	Unadjusted Prevalence of GDM (2019-2021)
India	0.53	0.80
Andhra Pradesh	0.00	0.63
Arunachal Pradesh	1.61	0.87
Assam	0.27	0.95
Bihar	0.92	0.60
Chhattisgarh	0.09	0.27
Goa	2.86	4.88
Gujarat	0.46	0.35
Haryana	0.66	1.08
Himachal Pradesh	0.96	1.02
Jharkhand	0.15	0.55
Karnataka	0.42	1.81
Kerala	3.41	3.06
Madhya Pradesh	0.52	0.69
Maharashtra	0.35	0.56
Manipur	0.15	0.00
Meghalaya	0.15	2.33
Mizoram	0.20	0.69
Nagaland	0.25	1.14
Odisha	0.35	0.43
Punjab	0.68	1.10
Rajasthan	0.10	0.42
Sikkim	0.00	0.00
Tamil Nadu	1.20	0.96
Tripura	0.00	1.64
Uttar Pradesh	0.41	0.63
Uttarakhand	0.44	0.45
West Bengal	0.77	1.04
Telangana	1.57	0.69

Source: Own calculations using NFHS (2015-16 and 2019-21) data

Note: Prevalence is per hundred women; *N*=30484 during 2015-16 and 26131 during 2019-21

To note, there was a substantial variation in the prevalence of GDM across the states of India in 2019–21. The state of Goa in western region and the state of Kerala in the southern region showed the highest prevalence of GDM, i.e.4.88 and 3.06%, respectively, in 2019–21. Following these states, Meghalaya northeastern region), Karnataka (southern region) Tripura and Nagaland (northeastern region), Punjab and Haryana (northern region), West Bengal (Eastern region) and Himachal Pradesh (northern region) showed a prevalence of GDM greater than 1%. Other states of India, showed a low prevalence of GDM; Manipur and Sikkim showed a 0% prevalence of GDM in 2019–21.

**Fig. 2:**
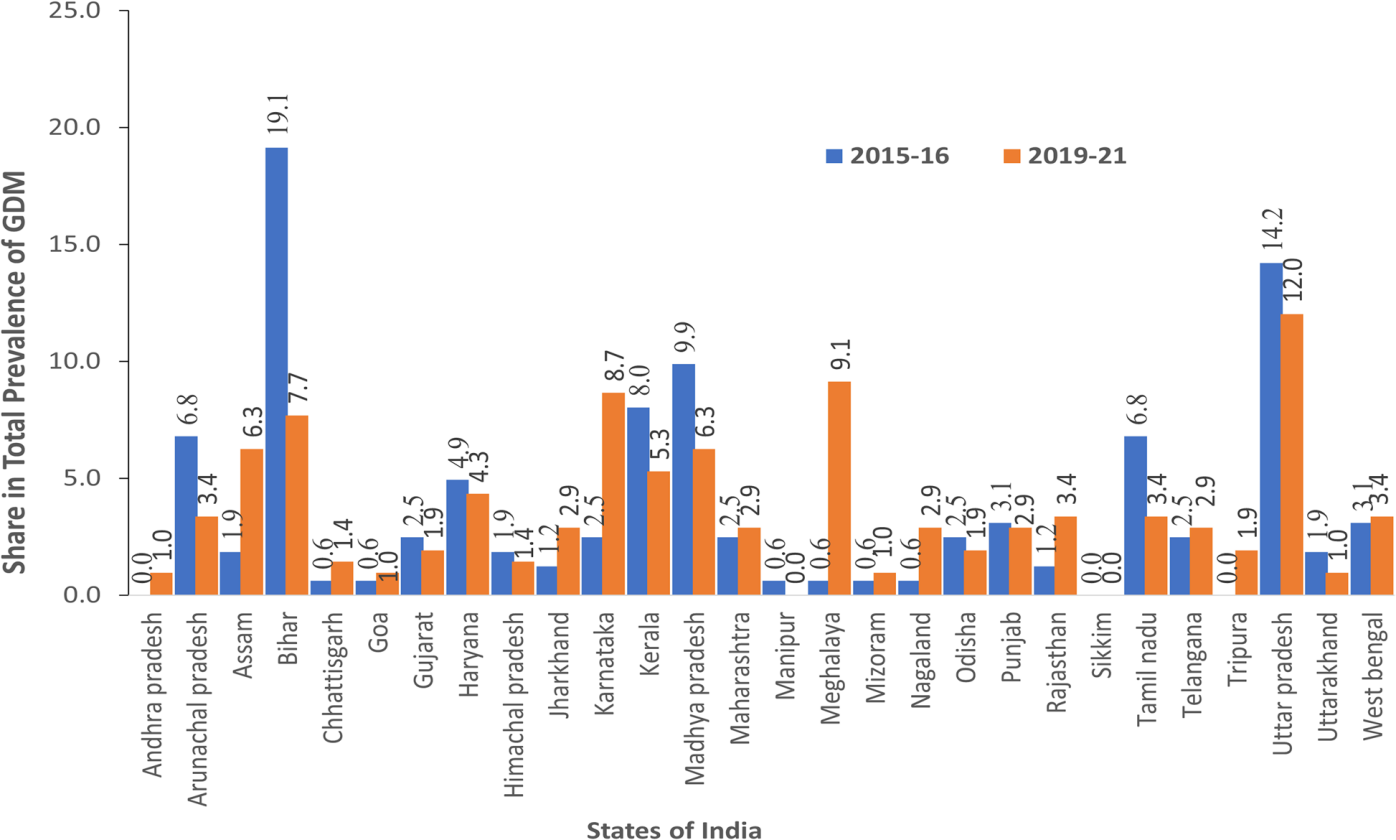
Change in the share of states of India in the total prevalence of GDM between 2015 and 16 and 2019–21

Figure 2 shows the change in the share of prevalence of GDM in states of India between 2015 and 16 and 2019–21. The states of Bihar, Uttar Pradesh, and Madhya Pradesh showed large shares of 19.14, 14.2 and 9.88% in the total prevalence of GDM in 2015–16. However, in 2019–21, the states of Uttar Pradesh, Meghalaya, and Karnataka showed large shares of 12.02, 9.13, and 8.65% in the total prevalence of GDM, respectively. Combined, these three states, namely, Bihar, Uttar Pradesh, and Madhya Pradesh, contributed 43.22% of the total cases of GDM in 2015–16, and Uttar Pradesh, Meghalaya, and Karnataka contributed 29.8% of the total cases of GDM in 2019–21. Following these states, Kerala and Tamil Nadu in the southern region, Arunachal Pradesh in the northeastern region, Haryana in the northern region, West Bengal and Odisha in the eastern region and Gujarat and Maharashtra in the western region showed large shares in the prevalence of GDM; combined, they showed a large share of 37.02% in the total prevalence of GDM in India in 2015–16. In sum, more than 80% of the total prevalence of GDM was shared by these states in the north, northeastern, eastern, western, and southern regions during 2015–16. Bihar and West Bengal in the eastern region, Haryana in the northern region, Arunachal Pradesh and Assam in the northeastern region, Madhya Pradesh in the central region and Kerala and Tamil Nadu in the southern region showed a large share of the total prevalence of GDM during 2019–21, and combined, they showed a share of 39.92% of the total cases of GDM in India in 2019–21. In sum, more than 65% of the total prevalence of GDM was shared by these states in the eastern, northern, northeastern, central and southern regions of India in 2019–21.

The results of the Poisson regression model are shown in Table 4. The deviance of the goodness of fit and the Pearson goodness of fit confirms that the Poisson regression model is a good fit for the sample data of GDM considered in the study. The Poisson regression model reveals that age group, BMI, heart disease, and thyroid disorder have a strong association with the prevalence of GDM in India. The Poisson regression coefficient for GDM was 0.67 for pregnant women in the age group of 25–29 years in reference to women in the age group of 15–19 years. This implies that keeping all other variables constant, with a progression in age, the prevalence of GDM is expected to be 0.67 times more in women aged 25–29 years than in women aged 15–19 years. Similarly, the prevalence of GDM is expected to be 1.00, 1.58, and 2.29 times higher in women in the age groups of 30–34, 35–39, and 40–44 years, respectively, than in women in 15–19 years, holding other variables constant in the model. Thus, with age progression, pregnant women have a higher prevalence of GDM.

**Table 4:** Poisson regression output to examine the possible risk factors for GDM, 2019-21, India

**Variables**	**Coefficient**	**Std. Error**	***z***	***p*** *> z*	**95% Confidence Interval (CI)**	
**Age groups**						
15-19 @						
20-24	0.133	0.307	0.43	0.67	-0.47	0.73
25-29	0.675	0.316	2.13	0.03	0.05	1.29
30-34	1.006	0.349	2.88	0.00	0.32	1.69
35-39	1.575	0.387	4.07	0.00	0.82	2.33
40-44	2.297	0.500	4.59	0.00	1.32	3.28
45-49	1.774	1.061	1.67	0.09	-0.30	3.85
**Level of education**						
No education @						
Primary	0.469	0.257	1.82	0.07	-0.04	0.97
Secondary	0.005	0.229	0.02	0.98	-0.44	0.46
Higher	-0.272	0.301	-0.90	0.37	-0.86	0.32
**Religion groups**						
Hindus @						
Muslims	0.226	0.206	1.10	0.27	-0.18	0.63
Christians	0.555	0.292	1.90	0.06	-0.02	1.13
others	-0.223	0.403	-0.55	0.58	-1.01	0.57
**Caste groups**						
SCs @						
STs	-0.476	0.278	-1.71	0.09	-1.02	0.07
others	0.510	0.267	1.91	0.06	-0.01	1.03
**BMI categories**						
Underweight @	0.000					
Normal	1.054	0.345	3.05	0.00	0.38	1.73
Overweight	1.174	0.373	3.15	0.00	0.44	1.90
Obese	1.599	0.414	3.86	0.00	0.79	2.41
**Children ever born**						
No child @						
One child	-0.109	0.175	-0.62	0.53	-0.45	0.23
Two children	-0.301	0.228	-1.32	0.19	-0.75	0.15
Three or more children	-0.673	0.278	-2.42	0.02	-1.22	-0.13
**Type of residence**						
Urban @						
Rural	0.168	0.196	0.86	0.39	-0.22	0.55
**Wealth Index**						
Poorest @						
Poorer	0.167	0.217	0.77	0.44	-0.26	0.59
Middle	0.306	0.226	1.35	0.18	-0.14	0.75
Richer	0.380	0.249	1.53	0.13	-0.11	0.87
Richest	0.117	0.305	0.38	0.70	-0.48	0.71
**Thyroid disease**						
No @						
Yes	1.295	0.253	5.13	0.00	0.80	1.79
do not know	-15.531	4756.408	0.00	1.00	-9337.92	9306.86
**Heart disease**						
No @						
Yes	2.328	0.354	6.58	0.00	1.64	3.02
do not know	-15.372	5015.501	0.00	1.00	-9845.57	9814.83
**Alcohol consumption**						
No @						
Yes	0.354	0.484	0.73	0.46	-0.59	1.30
**Smoking**						
No @						
Yes	0.330	0.278	1.19	0.24	-0.22	0.87
**Constant**	-6.775	0.503	-13.47	0.00	-7.76	-5.79

Source: Own calculations using NFHS 5 (2019-21)

Note: @ represents the reference category of a variable. N=26123. The chi-square test for the Poisson model was significant at 1% level of significance

Among religious groups, the expected change in the log count of the prevalence of GDM in Christian women increased by 0.55 times as compared to Hindu women. The coefficients of the Poisson regression model for women with normal weight, overweight, and obesity are 1.05, 1.17, and 1.60, respectively, compared with women with a below-normal BMI. Thus, the prevalence of GDM is largely associated with the body weight of women. Pregnant women with heart disease are expected to have a significant risk of elevated blood sugar levels during pregnancy. Pregnant women suffering from heart disease are expected to show an increased prevalence of GDM by 2.33 times as compared to pregnant women who are not suffering from heart disease. The coefficient of the Poisson regression model for GDM in pregnant women enduring a thyroid disorder is 1.29 in reference to pregnant women who do not have a thyroid disorder. Thus, the risk of having GDM in women enduring thyroid disorder increased by 1.29 times as compared to pregnant women not having thyroid disorder. Other socioeconomic and individual variables, such as caste (social group), level of education, wealth index, tobacco and alcohol consumption, children ever born and type of place of residence, did not show any significant association with the prevalence of GDM in women during pregnancy (Table 4).

## Discussion

### Summary of results

The study examined the prevalence of GDM in pregnant women in India by various background characteristics, including age, gender, urban city, social groups, and religion, using NFHS data from 2015 to 16 and 2019–21. Additionally, the study examined regional variation in the percentage prevalence of GDM in women by the states of India. This study analysed the determinants of GDM among pregnant women in India by the application of a Poisson regression model. A low prevalence of GDM qualifies as a rare disease. The change in the prevalence of GDM over time is apparent in urban areas, in Christians and Hindus, and in the age groups of 40–44 and 45–49 years. The gradient of the prevalence of GDM by age is most revealing and deterministic. The old-adult age group of 40–44 years in pregnant women showed a large prevalence of GDM at 2.2% in 2015–16, which increased to 3.9 per hundred pregnant women in 2019–21. Over time, the prevalence of GDM increased in childbearing age groups, with the exception of 15–19 and 25–29 years. An increase in the prevalence of GDM in urban areas and old-adult age groups indicates the consequences of an urbane lifestyle and is consistent with the onset of degenerative diseases. By caste, the prevalence of GDM was 1.53 and 0.55 per hundred pregnant women, respectively, in the general category and STs; over time, the prevalence remained unchanged in the categories of social groups.

There were substantial variations in the prevalence of GDM among the states of India in 2015–16 and 2019–21. Among the states of India, Kerala, Meghalaya, and Goa showed a large prevalence of GDM in 2015–16 as well as 2019–21. The change in the prevalence of GDM was evident in the states of West Bengal followed by Arunachal Pradesh, showing a large drop in the prevalence of GDM over time. Many states, such as Meghalaya, Goa, Tripura, Tamil Nadu, Karnataka, Nagaland, and Telangana, experienced an increase in the prevalence of GDM over time. However, a few states showed a decline in GDM over time. In sum, the prevalence of GDM showed an increase in many states that contributed to an increase in GDM at the national level from 0.66 to 0.79 per hundred pregnant women between 2015 and 16 and 2019–21. Among the states of India, the states of Uttar Pradesh and Bihar showed large shares of 17.8 and 10.6%, respectively, in 2019–21. Many states, such as West Bengal, Kerala, Tamil Nadu, Maharashtra, and Madhya Pradesh, showed a moderate share of the prevalence of GDM in the range of 5 to 10%. The results show that large shares of the prevalence of GDM were contributed by northern and eastern states of India.

The applied Poisson regression model on the prevalence of GDM at the national level with covariates attests to a strong association between age group, BMI, heart disease, and thyroid disorder. The Poisson regression model confirms a gradient in the prevalence of GDM by age, with the highest risk of GDM in the age group of 40–44 years. The Poisson regression model confirmed a significant association between BMI and the risk of GDM. Pregnant women in the normal BMI category showed a 1.05-fold higher risk than women in the low BMI category, and the gradient of GDM in pregnant women increased with higher BMI. The risk of GDM is expected to be higher in pregnant women enduring heart disease and thyroid disease.

### Discussion of results

The literature shows variation in the estimation of GDM prevalence in India, with most studies showing a somewhat higher prevalence, varying between 4 and 14%, than what is estimated from large-scale NFHS surveys [22, 30–32]. Variations in the estimate of GDM between NFHS surveys and small or specific surveys can be attributed to varying sample sizes and different regional settings in which the data are collected. Some studies adopted a different cut-off for measuring diabetes. Our study has taken into consideration the women who self-reported having diabetes in order to cover the entire sample. This can be a reason for the deviation in the prevalence of GDM calculated in our study from the study conducted on NFHS-4 data, which reported a GDM prevalence of approximately 1.3% [33].

Our study is one of the first to calculate GDM prevalence from NFHS-5 data. A study conducted on NFHS-4 data revealed that the prevalence of GDM is highest in the states of Kerala and Telangana [33], which is somewhat similar to our results conducted on NFHS-5. We also find Kerala among one of the states having a high prevalence of GDM in 2019–21. The prevalence of GDM was concentrated in the central and eastern states of Madhya Pradesh, Bihar, and Uttar Pradesh in 2015–16 [33]. Nonetheless, in 2019–21, NFHS data revealed that the prevalence of GDM was concentrated in the states of Uttar Pradesh, Meghalaya, and Karnataka. Women residing in rural areas have a higher prevalence of GDM than their urban counterparts; other (local) studies also reported a high prevalence of GDM in rural areas [20, 34]. Although not significant, urban women have a higher GDM prevalence than their rural counterparts, as evident in the study results. Higher GDM in urban areas compared to rural areas is also attested in a study conducted on the data from antenatal clinics in government primary health centres of Kancheepuram district and private maternity centres in Chennai city in Tamil Nadu between January 2013 and December 2014 [35].

Across the globe, many studies have found that increased BMI is a significant risk factor for GDM among pregnant women [27, 36–41]. A significant increase in age and BMI are risk factors for the prevalence of GDM [29, 42]. A few studies have also shown a differential in the prevalence of GDM by religion. Among the few, a study conducted on 50 pregnant women (ANCs) with GDM and 50 normal ANCs in Rajasthan to assess the biosocial demographic risk factors for GDM; they found that pregnant Muslim women are more prone to have GDM compared to Hindu women [43]. Similarly, studies conducted in rural Assam have also confirmed that women belonging to the Muslim religion have a higher likelihood of developing GDM during pregnancy [44]. However, the study, which was conducted on recent data from NFHS-5, contradicts the published results and shows that women belonging to Christian religions are more at risk of developing GDM during pregnancy. Our study has shown an association of thyroid disorder with the prevalence of GDM, which is in accordance with numerous studies that states GDM is associated with hypertension, hyperthyroidism, obesity, and lipid abnormalities [45]. GDM is considered a significant risk factor for long-term cardiovascular disease (CVD) in the follow-up period after pregnancy [46]. Another study on US women states that CVDs have a strong association with GDM. A study demonstrated that women with GDM are diagnosed with CVD approximately 23 years after the diagnosis of GDM [47]. Similarly, many other studies have shown a strong association between GDM and CVDs and stated that GDM is a risk factor for developing CVDs at a later stage of life [48, 49]. Our study is a prospective study that identified heart disease among pregnant women as a risk factor for developing GDM during pregnancy in India.

The absence of a dedicated nationwide program specifically addressing GDM in India represents a significant limitation in the current healthcare scenario. While existing maternal and child health programs play a vital role, they must not sufficiently cater to the unique challenges posed by GDM. This may result in gaps in awareness, early detection, and effective management of GDM, particularly for high-risk demographic groups such as older pregnant women and those with pre-existing health conditions. To address this, it is imperative to establish a comprehensive national program for GDM, incorporating awareness campaigns for healthcare providers and pregnant women alike. Emphasising research initiatives within the program is vital to continuously monitor GDM prevalence, identify high-risk populations, and assess the impact of interventions. By integrating GDM-specific components into existing health infrastructure and policies, India can mitigate these limitations, enhancing maternal and neonatal health outcomes across diverse populations. Such a targeted program would contribute not only to immediate improvements but also to the long-term health and well-being of mothers and their infants throughout the country.

### Limitation of results

The study is conducted on a large-scale national sample survey that has certain limitations. First, the data are self-reported data, and thus, various errors may be associated with it. Second, the nature of assessing diabetes in this survey limits the ability to distinguish between Type 1, Type 2, and GDM. Third, we restricted the study population to women who were pregnant at the time of the survey and assumed that diabetes reported by the pregnant women was GDM and not any preexisting disease or coincidental occurrence. An additional concern is that the study is predominantly quantitative, lacking the depth and context that qualitative research methods such as interviews and focus group discussion could offer. Incorporating qualitative approaches in future research would provide valuable insights into the experiences and perceptions of pregnant women, healthcare providers, and contribute a richer understanding of the challenges associated with gestational diabetes within the Indian healthcare landscape.

## Conclusion

The study identifies age, BMI, thyroid disorder, and heart disease as strong risk factors for GDM in Indian women. Additionally, it reports increased GDM prevalence across all age, religious, and caste groups (except ‘other’ castes) and types of residences.

India is a country with an increasing prevalence of diabetes. Gestational diabetes is a type of diabetes that is not only clinically significant but also has a high impact on families in particular and society at large. Mother suffering from diabetes increases the chances of DM in offsprings in their later years of life, giving birth to the cohort of future gestational mothers; also, if the disease is not controlled during pregnancy, then she herself may enter into the cohort of Type 2 diabetes in the future. Thus, considering the importance of this disease, it is essential to curb this disease since its inception and save a generation ahead.

### Supplementary Information

**Additional file 1:** **Fig. A1.** Changes in prevalence across age-group. **Fig. A2.** Change in prevalence across religious group. **Fig. A3.** Change in Prevalence across Social group. **Fig. A4.** Changes in prevalence across places of residence.

## Data Availability

The dataset supporting the conclusions of this article is available in the Demographic and Health Surveys (DHS) repository. The data can be downloaded from www.DHSprogram.com.

## Abbreviations

GDM: Gestational diabetes mellitus
DM: Diabetes mellitus
OGT: Oral glucose tests
NFHS: National Family Health Survey
DIPSI: Diabetes in Pregnancy Study Group in India
BMI: Body mass Index
GI: Glycaemic index
LSCS: Lower section caesarean section
CVD: Cardiovascular disease
SC: Scheduled Caste
ST: Scheduled Tribe
