# Supplementary material for: Prevalence and determinants of gestational diabetes mellitus among pregnant women in India: an analysis of National Family Health Survey Data
Source: BMC Womens Health. 2024 Feb 29;24:147. doi: 10.1186/s12905-024-02936-0 (PMC10902981; doi:10.1186/s12905-024-02936-0)
Supplement: Supplementary file 1 — Additional file 1: Fig. A1. Changes in prevalence across age-group. Fig. A2. Change in prevalence across religious group. Fig. A3. Change in Prevalence across Social group. Fig. A4. Changes in prevalence across places of residence. [file 12905_2024_2936_MOESM1_ESM.docx]

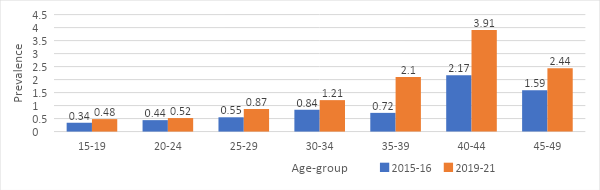


FigA1: Changes in prevalence across age-group


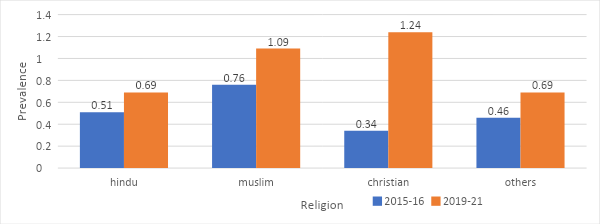


FigA2: Change in prevalence across religious group


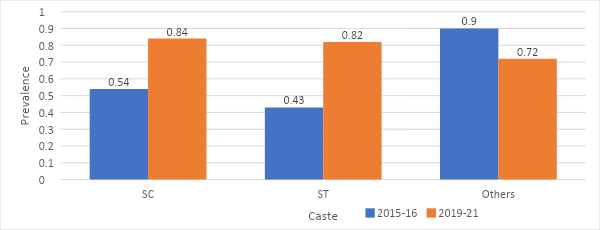


FigA3: Change in Prevalence across Social group


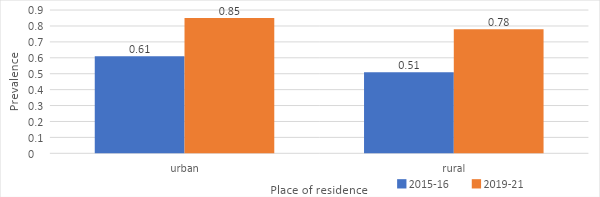


FigA4: Changes in prevalence across places of residence
